# Computational model links normalization to chemoarchitecture in the human visual system

**DOI:** 10.1126/sciadv.adj6102

**Published:** 2024-01-03

**Authors:** Marco Aqil, Tomas Knapen, Serge O. Dumoulin

**Affiliations:** ^1^ Spinoza Centre for Neuroimaging, Amsterdam, Netherlands.; ^2^ Computational Cognitive Neuroscience and Neuroimaging, Netherlands Institute for Neuroscience, Amsterdam, Netherlands.; ^3^ Experimental and Applied Psychology, Vrije Universiteit Amsterdam, Amsterdam, Netherlands.; ^4^ Experimental Psychology, Utrecht University, Utrecht, Netherlands.

## Abstract

A goal of cognitive neuroscience is to provide computational accounts of brain function. Canonical computations—mathematical operations used by the brain in many contexts—fulfill broad information–processing needs by varying their algorithmic parameters. A key question concerns the identification of biological substrates for these computations and their algorithms. Chemoarchitecture—the spatial distribution of neurotransmitter receptor densities—shapes brain function. Here, we propose that local variations in specific receptor densities implement algorithmic modulations of canonical computations. To test this hypothesis, we combine mathematical modeling of brain responses with chemoarchitecture data. We compare parameters of divisive normalization obtained from 7-tesla functional magnetic resonance imaging with receptor density maps obtained from positron emission tomography. We find evidence that serotonin and γ-aminobutyric acid receptor densities are the biological substrate for algorithmic modulations of divisive normalization in the human visual system. Our model links computational and biological levels of vision, explaining how canonical computations allow the brain to fulfill broad information–processing needs.

## Teaser

Mathematical models and multimodal neuroimaging show how neurotransmitter systems modulate cortical computations.

## INTRODUCTION

Computational models aim to provide an explicit mathematical description of the information processing carried out by the nervous system. The visual system has long been used as a beachhead for these approaches (*1*). Computations first identified in the visual domain have later been observed in other sensory modalities or cognitive domains (*2*–*9*).This has led to the proposal that some of these computations, such as receptive fields and divisive normalization (DN), may be “canonical,” i.e., that the same mathematical operations might be used by the brain in a variety of contexts (*10*, *11*). Depending on local parametrization of the algorithm (for example, the choice of weights in a receptive field), canonical computations can fulfill a variety of information-processing requirements and capture a wide range of seemingly disparate brain responses (*11*, *12*). A crucial question concerns the identification of biological mechanisms implementing and modulating the algorithms of these computations (*13*).

DN is considered a prime candidate for a canonical computation. Originally introduced to explain nonlinear properties of V1 neurons (*2*), it has since been applied in a variety of contexts (*11*). In vision, the population receptive field (pRF) is the region of visual space that elicits a response from a population of neurons (*14*, *15*). We have recently introduced a pRF model based on DN and shown that it unifies and outperforms existing pRF models by flexibly capturing a variety of brain responses (*12*). We have also shown that variations in specific model parameters explain different information-processing signatures observed throughout the visual hierarchy (*12*).

Chemoarchitecture—the spatial distribution of neurotransmitter receptor densities—is a major contributor to brain dynamics and functional properties in health and disease (*16*–*19*). Multiple lines of evidence suggest the involvement of neurotransmitter systems in neural computations. Electrophysiological studies in animal models have shown that neurotransmitter receptors modulate neuronal responses in visual areas (*20*–*25*). For instance, some receptors might influence stimulus sensitivity, by exerting differential modulations of response gain (e.g. 5-HT1B and 5-HT2A); others might influence integration of visual stimuli over space, by modulating relevant neuronal properties, such as baseline activity and inhibition [e.g. 5-HT1A and γ-aminobutyric acid (GABA)]. Clinical studies in humans have also found altered neurotransmission and altered information processing in several brain disorders (*15*, *26*–*29*). However, explicit links between chemoarchitecture and brain computations have not yet been clearly identified. A key step in this sense is the inclusion of an algorithmic level of description; lacking this, any approach can identify, but does not necessarily explain, relationships between biological and functional levels (*30*).

Here, we address this issue by bringing together explicit mathematical modeling of brain responses (*12*, *14*) with chemoarchitecture data (*31*–*33*). Bridging the gap between biological and functional levels of description, we hypothesize that spatially varying receptor densities in the human cortex modulate the algorithms of canonical computations. This would explain how the brain can produce a wide range of responses and achieve a variety information-processing goals with a single computation.

On the basis of existing neurophysiological evidence (*11*, *21*, *23*–*25*), we hypothesized the involvement of serotonin (5-HT1A, 5-HT1B, and 5-HT2A) and GABA receptors in DN. To test this hypothesis, we obtain algorithmic DN parameters from modeling of ultrahigh-field functional magnetic resonance imaging (fMRI) responses in a large cohort (*n* = 171) (*12*, *14*, *34*). We then compare DN computational model parameters with receptor density maps obtained from an independent positron emission tomography (PET) imaging experiment (*31*–*33*). This approach allows us to noninvasively investigate the algorithmic roles of receptor densities in cortical computations. We report a notable alignment between the fMRI-based computational model parameters and PET-based receptor density maps, consistent with our hypothesis. The pattern of association between the two independent datasets provides evidence in favor of the theoretical role of neurotransmitter systems as algorithmic modulators of canonical computations. In particular, we find strong support for the hypothesis that serotonin and GABA receptors modulate DN in the human brain.

## RESULTS

### DN pRF model links computational and biological levels of the human visual system

**Fig. 1.**
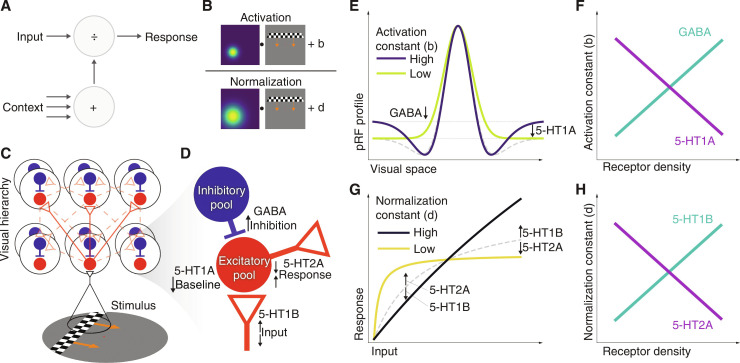
A model-based approach to visual computations and relationships between normalization and receptor densities. (**A**) The brain combines direct input and context to fulfill a variety of information-processing goals. DN is considered a prime candidate for this computation. (**B**) The algorithm of our DN pRF model (Eq. 1). The model describes responses to visual-spatial stimuli as the ratio of activation (input) and normalization (context). The modulation exerted by activation and normalization constants allows the DN model to capture a variety of information-processing signatures (*12*). At the implementation level, the DN model captures a combination of (**C**) circuitry, comprising feedforward, lateral, and feedback connectivity, and (**D**) receptors. (**E**) The activation constant of the DN model modulates the degree of suppression, shown in the difference between a pRF profile with strong suppression (dark blue line, high activation constant) and one with no suppression (green line, low activation constant). We hypothesize that inhibition and baseline activity may be relevant biological mechanisms for suppression. (**F**) Following this hypothesis, we predict that GABA and 5-HT1A receptor densities should have opposite correlations with estimates of the activation constant (positive and negative, respectively). (**G**) The normalization constant of the DN model (inversely) modulates the degree of compression, shown in the difference between an approximately linear response (black line, high normalization constant) and a strongly nonlinear (compressive) response (yellow line, low normalization constant). We hypothesize that modulations of input and response gain may be relevant biological mechanisms for compression. (**H**) Following this hypothesis, we predict that 5-HT1B and 5-HT2A receptor densities should have opposite correlations with estimates of the normalization constant (positive and negative, respectively).

**Fig. 2.**
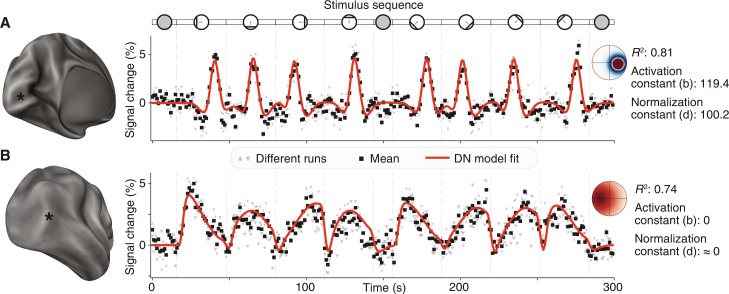
DN pRF model captures distinct information–processing signatures at different cortical locations. (**A**) Example data and model fits from an early visual cortex location (V1). The time course is characterized by low compression, visible in the similarly constant slope of the response profile to the bar passes, and high suppression, visible in the negative deflections of the blood oxygen level–dependent (BOLD) time course on the sides of all positive responses to bar passes. The former is captured in the DN model by a high value of the normalization constant, and the latter is captured in the DN model by a high value of the activation constant. (**B**) Example data and model fits from a late visual cortex location [Middle Temporal/Temporal Occipital (MT/TO)]. The time course is characterized by high compression, visible in the nonlinear response profiles to the bar passes, and low suppression, visible in the lack of negative, below-baseline deflections in the time course. The former signature is captured in the DN model by a low value of the normalization constant, and the latter is captured in the DN model by a low value of the activation constant. Insets: Cortical surfaces with asterisks indicating the respective location of the time courses (left), schematic of the stimulus sequence, comprising 8-bar passes in cardinal and diagonal directions (*34*) (top), two-dimensional pRF profile in the visual field, highlighting positive (red) and negative (blue) locations, model fit quality (*R*^2^), and estimates of DN model constants (right).

The properties of pRFs are robustly measurable with a variety of neuroimaging and recording methods (*5*, *15*, *35*, *36*). Distinct computational properties of pRFs, such as visual-field position, size, surround suppression, and compressive spatial summation (nonlinearity), have been observed, modeled, and shown to vary systematically throughout the human visual system (*12*, *14*, *37*, *38*). In this context, by surround suppression, we mean below-baseline deflections of time courses observed on the flanks of the central activation pRF, particularly prominent in early visual cortex (Figs. 1E and 2A) (*37*); by compressive spatial summation, we mean sublinear scaling of responses to increasing input, particularly prominent in late visual cortex (Figs. 1G and 2B) (*38*). We have previously shown that the DN model captures suppression and compression (as well as combinations of the two) via local variation in its algorithmic parameters, the activation, and normalization constants (Eq. 1 and Fig. 1B) (*12*). Here, following available evidence from neurophysiology (*21*, *23*–*25*), we hypothesize that serotonin and GABA receptors might represent the biological substrate for algorithmic modulations of visual-spatial DN computations. In particular, we hypothesize that different densities of these receptors might underlie the observed variations in suppressive information– and compressive information–processing signatures, captured in the DN model by the activation and normalization constants (Fig. 1, D, E, and G) (*12*).

Suppression, in the sense intended here, is likely to require inhibitory mechanisms, as well as an ongoing baseline activity that can be inhibited even in the absence of driving stimuli (*39*). Hence, mechanisms involved in the control of inhibition and baseline activity may be relevant biological correlates for suppression. Neurophysiological evidence suggests that GABA-mediated inhibition modulates responses to visual stimuli (*24*), while the 5-HT1A receptor has been recently shown to control baseline activity in rodent primary visual cortex (*23*). Hence, we hypothesize that different densities of GABA and 5-HT1A receptors might show opposite modulations of suppressive information–processing signatures (Fig. 1E). Following this hypothesis, we predict that the DN model activation constant should correlate positively with GABA receptor density and negatively with 5-HT1A receptor density in the visual system (Fig. 1F).

Compression implies relatively strong responses to weak input and relatively constant responses over a wide range of increasing input. Hence, mechanisms involved in the control of input and response gain may be relevant biological correlates for compression. Neurophysiological evidence suggests that 5-HT1B and 5-HT2A receptors implement a bidirectional, complementary modulation of input and response gain (*20*, *21*). Activation of the 5-HT1B receptor has been found to boost neuronal responses to strong stimuli, while dampening responses to weak stimuli, whereas the converse pattern has been observed for the 5-HT2A receptor (*20*, *21*). Hence, we hypothesize that different densities of 5-HT1B and 5-HT2A receptors might show opposite modulations of compressive information–processing signatures (Fig. 1G). Following this hypothesis, we predict that the DN model normalization constant should correlate positively with 5-HT1B receptor density and negatively with 5-HT2A receptor density (Fig. 1H).

### Population-level DN parameters are obtained from a large-scale fMRI dataset

**Fig. 3.**
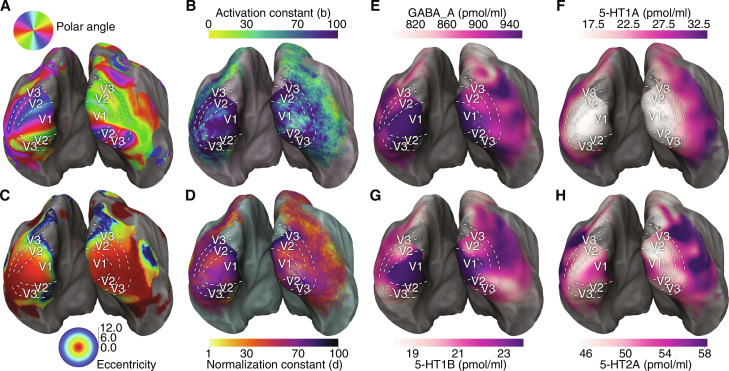
Estimates of DN model parameters and receptor density maps from independent fMRI and PET datasets. Cortical maps of classic retinotopic visual field parameters, (**A**) polar angle and (**C**) eccentricity, obtained by replicating the HCP fitting procedure (*34*). The DN pRF model recovers the known retinotopic features of visual field maps. Cortical maps of the DN model modulatory parameters, (**B**) activation constant and (**D**) normalization constant, obtained by fitting the model to the data of each HCP participant individually (*n* = 171). The parameters robustly and systematically vary throughout the visual system (see also fig. S1). (**E**) GABA, (**F**) 5-HT1A, (**G**) 5-HT1B, and (**H**) 5-HT2A receptor density maps in the visual system, obtained from PET imaging data (*31*–*33*). A first qualitative analysis already indicates that variations in receptor densities reflect known functional properties of the visual system. In particular, a marked drop in both GABA and 5-HT1B density is evident at the V1 border, while the two receptor differ in their variation at later stages of the different visual streams. The 5-HT1A receptor shows a systematic pattern of increasing density in the posterior-anterior direction, from early to late visual areas; while the 5-HT2A receptor shows a more complex pattern, with relatively high density in V1, decreasing in V2/V3, highest density in lateral-temporal areas, and lowest density in dorsal areas. Outlines of the first visual regions of interests (ROIs) displayed on all surfaces are based on the HCP–multimodal parcellation (HCP-MMP) atlas (see also fig. S2) (*42*).

To test our hypotheses, we obtained estimates of DN model parameters from the large-scale (*n* = 171) Human Connectome Project (HCP) retinotopy dataset (*34*, *40*). We then compare them with receptor density maps obtained by combining several independent PET imaging datasets (*31*–*33*). The DN model (*12*) recovers the well-known polar angle and eccentricity visual field maps throughout the human visual system (Fig. 3, A and C) (*41*). Fitting the DN model to each individual HCP participant data (Fig. 2) and averaging the resulting maps across participants, we also obtained group-average cortical maps of DN model activation and normalization constants (Fig. 3, B and D). We have previously shown that DN model constants systematically vary throughout the visual hierarchy, capturing the variation of suppression and compression across different visual areas (*12*). We replicate here our previous findings of systematic variation of DN model constants, in a larger cohort (fig. S1). For ease of visualization, we also show DN model parameter estimates in the visual system on flattened cortical surfaces (fig. S2).

### Receptor densities vary systematically throughout the human visual system

Visual inspection of receptor density maps (Fig. 3, E to H) reveals that they covary with the functional visual hierarchy (Fig. 3, E to H). In particular, both 5-HT1B and GABA receptors show high density in V1, dropping starkly at the V1/V2 border; however, 5-HT1B and GABA receptors differ in their variation in later areas, as GABA density drops to its lowest values in dorsal areas, remaining relatively high in ventral visual areas; whereas the 5-HT1B receptor density drops to low values both in the dorsal and ventral directions. Conversely, the 5-HT1A receptor density is lowest in low-level visual areas and increases systematically in the posterior-anterior direction, reaching its highest values in temporal and ventral regions. Last, 5-HT2A receptor density displays a more complex pattern of variation in the visual system, with medium-high density in V1, decreasing in V2 and V3. 5-HT2A receptor density then increases again, reaching highest density in temporal and lateral occipital regions, while instead decreasing to lowest density in late dorsal regions (Fig. 3, E to H). For ease of visualization, we also show receptor density maps in the visual system on flattened cortical surfaces (fig. S2).

### Receptor densities correlate with DN model constants

**Fig. 4.**
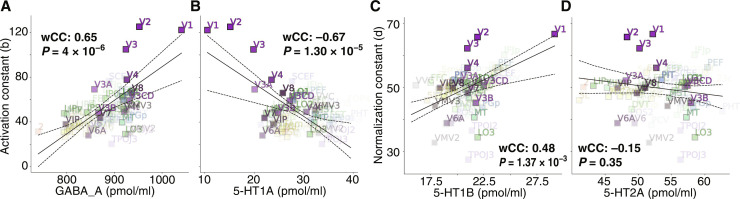
Activation and normalization constants correlate with serotonin and GABA receptor densities. (**A**) GABA receptor density correlates positively with the activation constant, consistent with our hypothesis. (**B**) 5-HT1A receptor density correlates negatively with the activation constant, consistent with our hypothesis. (**C**) 5-HT1B receptor density correlates positively the normalization constant, consistent with our hypothesis. (**D**) 5-HT2A receptor shows a nonsignificant negative correlation with the normalization constant. Continuous lines represent the best-fit linear regression; dashed lines represent 95% confidence intervals on the regression line, computed by bootstrapping. Statistical significance was assessed with two-sided Fisher’s permutation test (10^6^ permutations).

Next, we computed correlations between the model constants and receptor densities (Fig. 4 and Materials and Methods) across predefined regions of interests (ROIs) of the HCP–multimodal parcellation (HCP-MMP) atlas (*42*), shown on flattened cortex in (fig. S2). The GABA receptor density correlates positively with the activation constant, consistent with our hypothesis [weighted correlation coefficient (wCC): 0.65, *P* = 4 × 10^−6^; Fig. 4A]; the 5-HT1A receptor density correlates negatively with the activation constant, also consistent with our hypothesis (wCC: −0.67, *P* = 1.30 × 10^−5^; Fig. 4B). Further, as hypothesized, the 5-HT1B receptor density correlates positively with the normalization constant (wCC: 0.48, *P* = 1.37 × 10^−3^; Fig. 4C). The 5-HT2A receptor shows a nonsignificant negative correlation with the normalization constant (wCC: −0.15, *P* = 0.35; Fig. 4D). The relationship between the 5-HT2A receptor density and the normalization constant appears to follow a bimodal pattern. Early, temporal, and ventral ROIs appear to follow the hypothesized negative relationship, whereas a cluster of dorsal ROIs does not (Fig. 4D) (see Discussion).

In sum, we find a notable alignment between the independently estimated receptor density maps (PET) and DN model constants (fMRI). In particular, we find direct evidence in favor of the hypothesis that 5-HT1A and GABA receptor densities represent biological correlates of the activation constant (suppression). We also find direct evidence in favor of the hypothesis that the 5-HT1B receptor density might represent a biological correlate of the normalization constant (compression), while the role of the 5-HT2A receptor remains more ambiguous.

### DN model constants exhibit multidimensional relationships with receptor densities

**Fig. 5.**
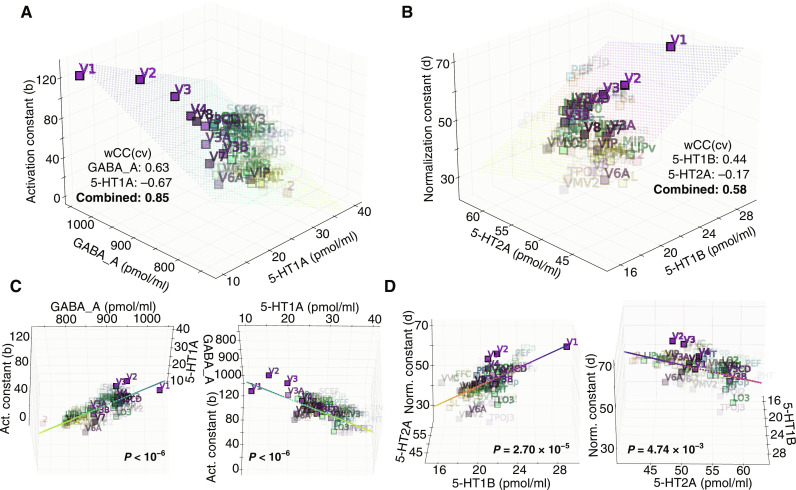
Receptor pairs provide bidirectional, complementary modulation of DN model constants. (**A**) The linear combination of GABA and 5-HT1A receptors provides a better cross-validated prediction for the activation constant compared to either GABA alone or 5-HT1A alone. This suggests that the distributions of both receptors combine to provide a bidirectional modulation of the activation constant. (**B**) The linear combination of 5-HT1B and 5-HT2A receptors provides a better cross-validated prediction for the normalization constant compared to either 5-HT1B alone or 5-HT2A alone. This suggests that the distributions of both receptors combine to provide a bidirectional modulation of the normalization constant. (**C**) In-plane views of the two-receptor GLM shown in (A), showing the variation of the activation constant as function of GABA and 5-HT1A receptors, after the other receptor in the pair has been taken into account. (**D**) In-plane views of the two-receptor GLM shown in (B), showing the variation of the activation constant as function of 5-HT1B and 5-HT2A receptors, after the other receptor in the pair has been taken into account. Statistical significance was assessed with two-sided Fisher’s permutation test (10^6^ permutations).

The one-dimensional correlations reported in Fig. 4 could be obscured by potential relationships between receptor distributions. To address this, we investigated whether the (linear) combination of receptor pairs that we hypothesized to be related to each DN model constant provides a better prediction compared to single receptors in the pair (Fig. 5).

We fit one-receptor and two-receptor general linear models (GLMs) on estimates of DN parameters obtained from one-half of the HCP participants and compare their predictions on parameter estimates obtained from the other, independent, half of participants, obtaining a cross-validated correlation coefficient wCC(cv). We find that the linear combination of GABA and 5-HT1A receptors provides a better cross-validated prediction for the activation constant [wCC(cv): 0.85] compared to either GABA alone [wCC(cv): 0.63] or 5-HT1A alone [wCC(cv): −0.67] (Fig. 5A). We also find that the linear combination of 5-HT1B and 5-HT2A receptors provides a better cross-validated prediction for the normalization constant [wCC(cv): 0.58] compared to either 5-HT1B alone [wCC(cv): 0.44] or 5-HT2A alone [wCC(cv): −0.17] (Fig. 5B). Last, we compare the two-receptor models with surrogate models comprising, in turn, one receptor in the pair and randomized permutations of the other (Fisher’s permutation test, 10^6^ permutations) (Fig. 5, C and D). In all four cases, we find that the two-receptor models with both true receptor structures in the pair perform better than surrogate models with one true and one randomized receptor, in a statistically significant way (GABA, *P* < 10^−6^; 5-HT1A, *P* < 10^−6^; 5-HT1B, *P* = 2.70 × 10^−5^; 5-HT2A, *P* = 4.74 × 10^−3^). This result indicates that the improved performance of the two-receptor models is due to the combined spatial structure of the receptors in the pair and not simply obtainable by adding a regressor with an equivalent distribution of values. Notably, this result goes beyond the pairwise comparison results above by showing that the 5-HT2A receptor density also provides a plausible biological correlate for the normalization constant, consistent with our hypothesis, provided that the spatial distribution of the 5-HT1B receptor density is taken into account (Fig. 5D).

In sum, the multidimensional analysis consolidates the evidence for the hypothesized relationships between receptor densities and DN model constants. In particular, the combination of both receptors provides better cross-validated predictions for DN model constants, above and beyond individual receptors; statistical analysis confirms that the improvement is specific to the true spatial structure of receptor densities. Hence, these results provide additional evidence for the involvement of the hypothesized neurotransmitter systems in visual-spatial DN computations. Our findings indicate that GABA and 5-HT1A might represent independent and complementary correlates of the DN model activation constant and that 5-HT1B and 5-HT2A might represent independent and complementary biological correlates of the DN model normalization constants.

### Principal components of receptor dataset correlate with DN model constants

**Fig. 6.**
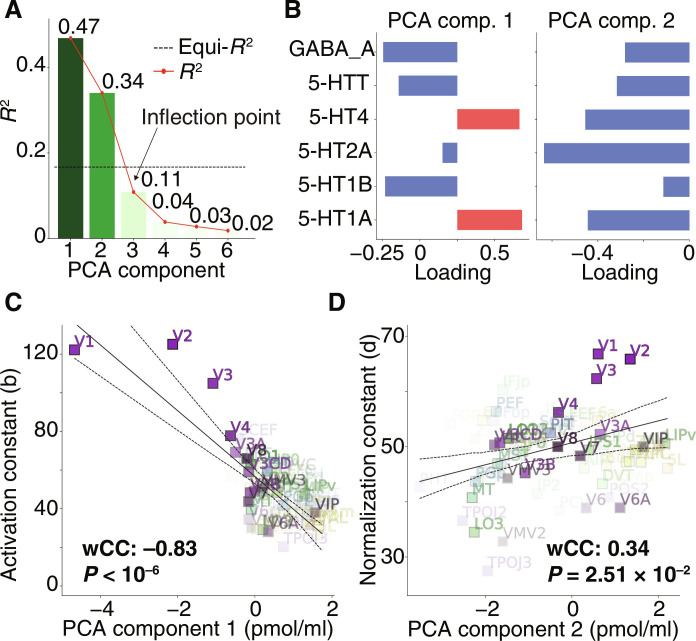
PCA provides additional evidence in favor of hypothesized relations between receptor density maps and DN model constants. (**A**) Variance explained (*R*^2^) by principal components of the receptor dataset. We include in the PCA all receptors available in the dataset, even those for which we lack a specific hypothesis (5-HT1A, 5-HT1B, 5-HT2A, 5-HT4, 5-HTT, and GABA). (**B**) Composition of the first two PCA components of the receptor dataset, which together account for 81% of the variance. The first component receives its largest contributions from GABA, 5-HT1B, and 5-HT1A receptors, with the 5-HT2A providing the smallest contribution; conversely, the second component receives its largest contribution from the 5-HT2A receptor. (**C**) The first PCA component has its largest correlation with the activation constant, out of all DN model parameters. (**D**) The second PCA component has its largest correlation with the normalization constant, out of all DN model parameters. This data-driven analysis confirms the notion that neurotransmitter systems are involved in modulating visual-spatial computations and, in particular, that they might represent biological correlates of the DN model activation and normalization constants.

Last, our hypothesis-driven approach may have overlooked other relationships between receptor density maps and DN model parameters. To examine this possibility in a data-driven way, we conducted a principal components analysis (PCA) of the receptor dataset and correlate these principal components with DN model parameters. For generality, we include all available receptors and all DN model parameters, including those for which we lack specific hypotheses. The full receptor dataset includes 5-HT1A, 5-HT1B, 5-HT2A, 5-HT4, 5-HTT, and GABA receptor density maps (*31*–*33*). We find that the receptor dataset is characterized by two major principal components, the key directions of variation in the receptor dataset. These two components together account for 81% of total variance and are the only components above equivariance threshold (Fig. 6A). The first component receives its largest contributions from GABA (negative), 5-HT1B (negative), and 5-HT1A (positive) receptor densities, with the 5-HT2A providing the smallest contribution. The second component receives same-sign contributions from all receptors, with the largest being the 5-HT2A receptor density and the smallest being the 5-HT1B receptor density (Fig. 6B).

Next, we computed correlations between the two major PCA components of the receptor dataset and each of the DN model parameters (Eq. 1), i.e., activation and normalization pRF sizes (σ_1_ and σ_2_), eccentricity, polar angle, activation and normalization pRF amplitudes (*a* and *c*), and activation and normalization constants (*b* and *d*) (fig. S3). The first PCA component shows significant correlations with several DN model parameters; its strongest correlation is with the activation constant *b* (wCC: −0.83, *P* < 10^−6^) (Fig. 6C). This result is notably consistent with our initial hypotheses: The first PCA component receives major contributions, opposite in sign, from GABA and 5-HT1A receptor densities and indeed correlates with the activation constant. The first component also receives a major negative contribution from the 5-HT1B receptor and indeed also significantly correlates negatively with the normalization constant (wCC: −0.62, *P* = 6 × 10^−6^; fig. S3). The second PCA component is dominated by the 5-HT2A receptor, which is almost entirely absent from the main first component, suggesting a unique role of the 5-HT2A receptor in the visual system, orthogonal to the main component of variation. Notably, the normalization constant has the strongest correlation of all DN model parameters with this second PCA component (wCC: 0.34, *P* = 2.51 × 10^−2^) (Fig. 6D). The direction of correlation is again consistent with the hypothesized relationship between 5-HT2A receptor and the normalization constant: The second PCA component receives a major (negative) contribution of 5-HT2A and indeed correlates with the normalization constant. Similarly to the relationship between the 5-HT2A receptor density and the normalization constant (Fig. 4D), this relationship appears to be bimodal; an association pattern is present in the combination of early, ventral, and temporal ROIs, while a cluster of dorsal ROIs follows a different pattern.

In sum, a PCA of receptor density maps provides additional evidence for the involvement of neurotransmitter systems in the suppressive and compressive modulation of DN computations throughout the visual system, in particular, for GABA and 5-HT1A as correlates of the activation constant and for 5-HT1B and 5-HT2A as correlates of the normalization constant.

## DISCUSSION

By integrating explicit mathematical modeling of brain responses with chemoarchitecture data and drawing hypotheses from neurophysiological literature, here, we provide a new approach to bridging the gap between biological and computational levels of vision (*30*). We focus on DN, in the context of visual-spatial pRFs, to provide the link between these levels. In particular, following existing neurophysiological evidence, we hypothesized that the DN model’s activation and normalization constants, which respectively capture suppressive information– and compressive information–processing signatures, might be explained by underlying variations in different receptor densities. To test these hypotheses, we compared estimates of DN computational model parameters obtained from a large-scale fMRI dataset, with receptor density maps obtained from an independent PET imaging experiment. We find evidence in favor of the hypotheses that 5-HT1A and GABA receptor densities are associated with the DN model’s activation constant (suppression) and that 5-HT1B and 5-HT2A receptor densities are associated with the DN model’s normalization constant (compression). These findings are supported by multiple analyses, thereby providing evidence of how varying receptor densities can allow the brain to fulfill a variety of information-processing requirements with a single canonical computation.

Primary visual cortex (V1) is a major contributor to visual processing. V1 is an area serving a unique role in vision, both biologically and computationally, as the first cortical retinotopic area. On the biological side, V1 is the visual area with the largest surface area and has the highest/lowest densities for several receptors. In particular, the 5-HT1B receptor density drops sharply at the V1/V2 border (Fig. 3G). On the computational side, V1 has the highest DN model *cvR*^2^, which implies that its role is amplified in weighted correlations. It also has the highest or lowest values for several DN model parameters. We contend that these estimates should not be considered statistical outliers, i.e., an extreme value due to noise fluctuations of a uniform or normal distribution; rather, the extrema of DN parameters and receptor densities observed in V1 reflect its functional and biological uniqueness. The fact that independent datasets of DN model parameters and receptor densities both show extreme values in V1 provides additional support to the correspondence between DN computations and the underlying biology. To address the role of V1 in the relationships we identified, we repeated the analyses of Figs. 4 and 5 but in the absence of V1. We find that the results are near-identical, showing that the identified correlations are robust throughout the visual system, even in the absence of V1 (figs. S4 and S5).

Here, we compute correlations across predefined ROIs of the HCP-MMP atlas (*42*). We do this because (a) the effective resolution differs between the two datasets, complicating comparisons at the vertex level; (b) different ROIs are expected to be functionally distinct, and, hence, variation across ROIs should reflect global computational diversity, whereas we assume that similar computations exist within each ROI; (c) ROI-wide estimates provide more robust measures of both receptor densities and DN model constants than vertex-wise, as the latter suffer more the confounds due to warping and resampling, intrinsic in neuroimaging data; (d) for retinotopically defined ROIs such as V1, V2, or V3, within-ROI variance is by definition dominated by eccentricity, and, hence, vertex-wise correlations may come to include eccentricity-driven effects, unrelated from the hypotheses that we are testing here; (e) different spatial autocorrelations in the two types of data (DN parameters versus receptor densities) will affect statistical outcomes in ways that are difficult to fully control; and (f) consistency with previous studies of neurotransmitter receptors that also use ROI-wide estimates. Nevertheless, we acknowledge that DN model constants and receptor density maps may also vary within ROIs. To account for this, we also compute correlations on the vertex-wise data, with a dedicated approach to control for spatial autocorrelations (note S1). We find the results are near-identical, showing that the correlations are robust regardless of the choice of computing ROI-wide or vertex-wise approach (fig. S6).

Our primary results here are obtained by means of between-ROI correlations. In this context, within-ROI correlations may also of potential interest. However, investigating within-ROI correlations presents several limitations and confounds. In chiefly retinotopic ROIs such as V1, V2, and V3, within-ROI variance is primarily driven by eccentricity. Hence, any identified within-ROI correlation between receptor densities and other model parameters is likely confounded by eccentricity-driven variation. A theoretically viable solution to this confound could be to parcellate a retinotopic ROI by coarse eccentricity (e.g., foveal, parafoveal, and peripheral) and investigate correlations within each parcel. This approach is challenged by the limited stimulus extent of the HCP fMRI experiment and the relatively low spatial resolution and smoothing of PET data. The stimulus subtends 8° of visual angle, and, hence, we cannot obtain reliable estimates of DN model parameters beyond this eccentricity. This eccentricity range corresponds to a few centimeters of V1 surface; any subparcellation applied in this range quickly runs into the spatial resolution limits of PET data. In addition, the variation of DN model parameters within few centimeters of the same ROI is relatively small, since most of the relevant functional variation is between ROIs. Nonetheless, we note that our PCA (Fig. 6 and fig. S3) and our vertex-wise correlation analysis (fig. S6) do take into account within-ROI heterogeneity. The agreement of these analyses with our main results shows that the between-ROI analysis is robust with respect to within-ROI heterogeneity. Last, we note that in this work, we have taken a hypothesis-driven approach, informed by neurophysiological findings, to disentangle the wealth of potential associations between the two datasets. This approach does not allow us to exclude additional relationships between other pRF parameters and receptor densities. Eccentricity is certainly an interesting dimension in this sense. One noteworthy observation that we have not included in our main findings, since it was not part of our hypotheses, is a robust and statistically significant positive correlation between the 5-HTT (serotonin transporter) receptor density and visual eccentricity (fig. S8). This finding may be of potential functional relevance and worth of future investigation.

The association between the 5-HT2A receptor and the normalization constant is supported by the multidimensional and data-driven approaches. In particular, combining 5-HT1B and 5-HT2A receptor densities provides a better (cross-validated) prediction for the normalization constant compared to the 5-HT1B receptor alone, and the contribution of the 5-HT2A receptor is statistically significant compared to surrogate models comprising 5-HT1B and randomized permutations of the 5-HT2A receptor density (Fig. 5). In addition, the second PCA component of the receptor dataset receives the highest contribution from the 5-HT2A receptor and is found to have the highest correlation with the normalization constant out of all DN model parameters (Fig. 6). We note that the relationship between the 5-HT2A receptor density and the normalization constant appears to follow a bimodal distribution. The relationship appears to follow our hypothesis when considering early, temporal, and ventral areas of the visual system; a cluster of dorsal visual areas exhibits a quite different pattern of results. We speculate that the role of the 5-HT2A receptor in visual computations might differ across different visual streams. Specifically, the 5-HT2A receptor might play the role of the hypothesized visual-spatial normalization constant in lower, temporal, and ventral areas, whereas it might serve a different computational role in dorsal areas. We speculate that datasets involving richer stimuli, for example, probing temporal and multisensory aspects (*43*, *44*) of DN, may be needed to disentangle the computational role of the 5-HT2A receptor throughout the human visual hierarchy.

There are many mathematical formulations of DN that have been used in the literature (*11*, *45*). For example, some authors included additional parameters for exponentiation of activation and normalization components, while others do not. We argued that our choice of formulation strikes a fruitful balance between interpretability and parsimony (*12*). Nonetheless, we acknowledge that an equivalent model formulation can be obtained, without loss of generality, by fixing one of the four parameters *a*, *b*, *c*, and *d* to an arbitrary constant. For example, by setting *d* = 1 in the model Eq. 1. This formulation is advantageous in terms of parsimony, since it has one fewer parameter, but misses the straightforward interpretability of the normalization constant as modulator of compression. Estimates for the *a*, *b*, and *c* parameters in the *d* = 1 formulation are identical to those in our formulation, up to a divisive factor, i.e., the value of *d* at that cortical location. We find that the estimate of the activation constant *b* in this reduced *d* = 1 formulation (i.e., *b*/*d* in our formulation) still presents a positive correlation with GABA receptor density (wCC: 0.71, *P* < 10^−6^) and a negative correlation with 5-HT1A receptor density (wCC: −0.59, *P* = 9.7 × 10^−5^) (fig. S7). Correlations for the normalization constant cannot be computed, since the parameter is kept fixed to a constant value *d* = 1. This result proves that the proposed relationships between chemoarchitecture and DN model parameters are not exclusive to a single formulation and, for the remaining parameter (activation constant), can also be obtained in a more parsimonious alternative formulation.

The stimulus used in the HCP dataset does not vary in contrast, as is usually done to estimate DN models’ neurophysiological contrast-response curves (*11*). Unlike these models, the DN pRF model aims to capture nonlinearities in visual space, e.g., compressive spatial summation (*38*), rather than in contrast. The standard moving-bar stimulus is shown at a variety of visual-field positions, orientations, and directions of motion (*14*). Hence, each cortical location is sampled with a variety of stimulus intensities due to its pRF position and size preferences. This process is akin, in space, to sampling at different contrast intensities for a contrast-response DN model. In a previous study (*12*), we varied bar sizes and speeds and found the same pattern of results in the DN parameters as with a single moving-bar stimulus. Whether the neural mechanism of the contrast-response nonlinearity is the same as spatial nonlinearity is an interesting question for future research but beyond the scope of this paper.

Our approach presents a number of methodological and conceptual limitations that should be emphasized. We are subject to the limitations of blood oxygen level–dependent (BOLD) fMRI. The BOLD signal does not allow distinguishing the activity of underlying neuronal subpopulations but only provides a proxy for overall activation, or deactivation, of the local neuronal population (~10^6^ neurons), relatively (in our case) a stimulus-free baseline. Hence, here, we cannot distinguish the contributions of individual neurons or different cell types. Nonetheless, we argue that the scale of neuronal populations is an appropriate scale for description of computational processes such as spatial vision, which rely on the combined activity of large numbers of neurons. This approach is complementary to neurophysiological approaches, which provide high specificity and sensitivity in probing single-neuron responses at the expense of spatial coverage and overall response. We are subject to the limitations of PET. PET imaging on its own does not provide information as to how receptor densities assayed with radioactive ligands map onto underlying neuronal and receptor function nor to the particular cortical layer or cell type where they would be found. A strength of our approach is indeed the possibility of connecting PET-measured receptor densities to computations. As our interest here lies with the computations performed at the level of large neuronal populations, as measured by fMRI, we believe PET is the measurement method that is most apt for comparison with fMRI results. Alternative methods to measure receptor densities exist, such as postmortem mRNA expression. The receptor density maps we use here have been compared to mRNA measures and found the be in agreement throughout the cortex (*31*, *32*). Approaches focusing on modeling single-neuron responses might instead benefit from more fine-grained measurements of receptor densities, such as mRNA expression. We are limited by existing datasets to analyzing group-level relationships between visual computations and chemoarchitecture. However, pRF parameters and receptor densities are known to vary across individuals (*31*, *46*). Future work using PET and fMRI in a single cohort of participants might be able to address whether the same associations can be observed at the individual level. However, this approach is limited by the health implications of PET neuroimaging. A complementary direction aimed at answering these questions could involve the administration of neuropharmacological agents known to be active at specific receptors and measurement of resulting changes in pRF parameters. We only investigate a computational DN model of spatial vision. Hence, our approach is limited to the brain areas where, given the signal-to-noise ratio of the available fMRI data, we are able to robustly measure and model responses to visual-spatial stimuli (here, approximately one-sixth of the entire cortical surface). The relationships that we identify here need not generalize to different models, different sensory modalities, or other brain areas. Nonetheless, DN is considered a prime canonical computation candidate and has been identified in a variety of contexts, and pRF modeling approaches have been used in different sensory and cognitive modalities. Hence, our results beg the question of whether the relationships between biological systems and computational processes presented here are limited to spatial vision or apply more widely throughout the brain. We only investigate receptors that sit at the intersection of those with openly available PET data and those for which hypotheses can be formulated on the basis of existing neurophysiological evidence. Our approach is not exhaustive; other receptors beyond those investigated in this study are known to be involved in visual computations. Most notably, acetylcholine and *N*-methyl-d-aspartate receptors (*47*–*50*). We cannot exclude that the information-processing signatures that we investigate here might involve additional or alternative mechanisms. Furthermore, it is certainly likely that the receptors that we investigate here are also involved in other kinds of computations (for example, on the basis of current results, we have speculated that the 2A receptor might subserve a different functional role in dorsal visual areas, compared to the rest of the visual system) and nonvisual processes throughout the brain. Our results also square well with studies on the receptor-nonspecific effects of serotonin in macaque visual cortex [e.g., (*22*)]. The administration of serotonin results in subtractive and divisive change to visual response profiles, without affecting tuning preferences. These observation are thought to reflect a net effect between facilitation and suppression, mediated by different receptor classes all activated by serotonin. Our approach is inherently correlational. Our hypotheses are formulated on the basis of the findings of neurophysiological experiments in animal models, which generally involve direct, causal manipulation of neurotransmitter receptors via optogenetic or pharmacological interventions. Our findings hint at strong relationships between the results of computational modeling and biological systems and would be cemented by investigations with a causal approach, for example, by applying a direct pharmacological intervention in humans that would directly engage some of the neurotransmitter receptors that we find here to be associated with computational model parameters of DN.

In sum, in this work, we propose that spatially varying receptor densities contribute to the range of information-processing signatures observed throughout the human visual hierarchy and captured by the computational parameters of DN, activation, and normalization constants. We formulate hypotheses and predictions based on neurophysiological evidence and test them by combining explicit mathematical modeling of brain responses and chemoarchitecture data. Our results support the hypothesized roles of neurotransmitter receptors as algorithmic modulators of canonical computations in the human brain. In particular, we find that 5-HT1A and GABA receptor densities show opposite correlations with the DN model activation constant, indicative of suppressive information–processing signatures, while 5-HT1B and 5-HT2A receptors show opposite correlations with the normalization constant, indicative of compressive information–processing signatures. By providing an explicit link (the DN pRF model and its algorithmic parameters) between biological (receptor densities) and computational (suppressive information– and compressive information–processing) levels of description, our results provide insights into brain function and open routes for computational neuropharmacology in the human brain.

## MATERIALS AND METHODS

### fMRI data acquisition and preprocessing

We obtain fMRI data from the HCP dataset, after it has been preprocessed according to HCP procedures and projected to a standard space (*34*). The HCP dataset comprises 7-T fMRI data for 171 participants. fMRI scans were collected, while participant viewed visual-spatial stimuli and performed an attention task at fixation. The stimulus conditions consisted of (a) a bar moving through the screen in eight different direction, (b) a clockwise rotating wedge, (c) a counterclockwise rotating wedge, (d) an expanding ring, and (e) a contracting ring. For each participant, two identical runs were collected for the moving-bar conditions; for all other conditions, only one run was collected. For additional details, see (*34*). Here, we use group-average time courses from all conditions to replicate the HCP-style analysis and show that the DN model recovers well-known eccentricity and polar angle maps; we use individual-participant data, from the moving-bar conditions only, to construct group-average maps of DN model parameters, since (a) the presence of two identical runs for the moving-bar condition enables the calculation of cross-validated model performance, which cannot be carried out for the other stimulus conditions; (b) when not carrying out cross-validation, averaging of at least two time courses markedly increases signal-to-noise ratio; and (c) wedges and rings are highly stereotyped stimulation pattern that do not allow probing pRF properties with the level of specificity enabled by the moving-bar stimulus, which, as it approaches receptive fields from a variety of directions, provides a more diverse set of responses and more robust estimation of pRF properties.

### PET data acquisition and preprocessing

We obtain receptor density maps from the Neurobiology Research Unit (NRU) atlases (*31*–*33*). PET imaging is used to compute quantitative density maps for 5-HT1A, 5-HT1B, 5-HT2A, 5-HT4, 5-HTT, and GABA receptors. For details, see (*31*–*33*).

### Model fitting

The DN model is defined as (*12*)

$$p_{\text{DN}}\left(t\right)=\frac{a\;G_{1}\cdot S+b}{c\;G_{2}\cdot S+d}-\frac{b}{d}$$ (1)

In the above equation, the spatial dependence of Gaussian pRFs *G* ≡ *G*(*x*, *y*) and the spatiotemporal dependence of stimuli *S* ≡ *S*(*x*, *y*, *t*) are omitted for brevity, and we denote

$$G_{1,2}=e^{-\frac{{\left(x-x_{0}\right)}^{2}+{\left(y-y_{0}\right)}^{2}}{2\sigma_{1,2}^{2}}},G_{1,2}\cdot S\equiv \sum_{x,y}‍\left(G_{1,2}\circ S\right)$$

Hence, (*x*_0_,*y*_0_) is the response central location in the visual field, σ_1_ and σ_2_ are the sizes of activation and normalization pRFs, *a* and *c* are their amplitudes, and *b* and *d* are the activation and normalization constants. The DN model is fit following the same procedure as (*12*). Briefly, the original Gaussian pRF model is fit, with a grid and iterative stage, to obtain initial estimates of pRF size and position. These estimates are used as starting parameters for the DN model fit, which also comprises a grid and iterative fit stages. First, a grid stage is performed vertex-wise over the additional DN model parameters, while keeping the pRF size and position fixed to the Gaussian estimates; next, an iterative fit over all parameters is performed, including pRF size and position. At the iterative fit stage of both Gaussian and DN models, we fit a one-parameter hemodynamic response function based on the scanning probe microscope basis set. This allows adjusting for the potentially different speed of the hemodynamic response across areas and participant. Fitting the hemodynamic response function speed also ensures that our results are not due to different hemodynamic response properties but reflect distinct information–processing signatures of different areas.

### HCP analysis replication

Following the same steps of the HCP analysis (*34*), we concatenate fMRI time courses for all available stimulus conditions (wedges, rings, and bars) and average data across participants. We carry out pRF model fitting on this population-averaged time courses to obtain estimates of classic visual field maps of polar angle and eccentricity.

### Population-level cortical maps of DN model parameters

We first obtain single-participant maps of DN model parameters by fitting the DN model to the mean of the two available bar-pass stimulus runs, for each participant, at each cortical location. Averaging the two runs ensures high signal-to-noise ratio, required for robust parameter estimates. However, the variance explained (*R*^2^) obtained from this fit is not cross-validated and therefore subject to potential overfitting issues. Hence, it cannot be taken as an unbiased measure of model performance. To address this, we compute the cross-validated variance explained (*cvR*^2^), as follows. We fit the model on each data run separately, evaluate performance on the other, independent run, and average the resulting two values. This gives us single-participant maps of *cvR*^2^, which provide an unbiased measure of model performance for each participant, at each cortical location. Next, we compute population-level parameter maps as the average of single-participant maps, weighted by the respective *cvR*^2^. For each participant, only cortical locations with a *cvR*^2^ > 0 are included because negative values cannot be used as weights and because model fits with a *cvR*^2^ < 0 cannot be considered reliable as they are likely the results of overfitting or highly noisy data. To obtain a population-level map of model fit quality, we compute the mean *cvR*^2^ across the participants with *cvR*^2^ > 0 at each cortical location. As these maps provide us with a measure of confidence in the parameter estimates, we use it for weighting in all subsequent analyses. Note that since a potentially different number of participants may have a *cvR*^2^ > 0 at each cortical location, population-level parameter maps may receive contributions from a different number of participants at each cortical location. To take this into account, we only include, in subsequent analyses, those cortical locations where the map resulted from averaging of least a minimum number of participants, i.e., cortical locations where the number of participants with *cvR*^2^ > 0 was higher than a minimum threshold value. To obtain this threshold in a data-driven way, we use Lins cross-entropy, a method often used in image processing for image segmentation/binarization. For our data, Lins cross-entropy gives a threshold value of 27.3 participants. Hence, we include in population-level parameter maps and subsequent analyses only cortical locations where at least 28 participants had a *cvR*^2^ > 0.

### Evaluating one-dimensional correlations between receptors and DN model constants

One-dimensional correlations are quantified using the (weighted) correlation coefficient, where the weight corresponds to the average *cvR*^2^ in that ROI. This ensures that the importance of data points in the correlation coefficient computation is proportional to the confidence in parameter estimates. We assess statistical significance of each one-dimensional correlation by building a specific null distribution and performing a two-sided Fisher’s permutation test. To do this, we obtain a null distribution for the independent variable by computing 10^6^ randomized permutations of its true observed values; for each permutation, we compute the weighted correlation with the true dependent variable. The (two-sided) *P* value is then computed as the fraction of permutations in the null distribution that have a correlation greater in magnitude than the weighted correlation between the dependent variable and the true independent variable. This procedure provides the probability that the correlation between the true independent variable (here, receptor density) and the true dependent variable (here, DN model constant) could also have been obtained with a “surrogate” independent variable, with the same distribution of values as the true one but randomized spatial structure.

### Evaluating two-receptor models

To compare two-receptor and one-receptor models, we use a cross-validated, weighted correlation coefficient. We fit one-receptor and two-receptor GLMs on maps of DN model constants obtained from one-half of all participants and evaluate the models on the other, independent half. Cross-validation ensures that two-receptor model do not outperform one-receptor models purely due to overfitting and allows a fair comparison between models with different number of parameters. We assess statistical significance of partial correlations of each receptor in the two-receptor models by building a specific null distribution and performing a two-sided Fisher’s permutation test. To do this, we compute 10^6^ randomized permutations of the true observed values of each receptor in the pair, while maintaining the true values of the other receptor in the pair; for each permutation, we fit a two-dimensional GLM to the true values of the DN model constant and compute the weighted correlation coefficient. The (two-sided) *P* value is then computed, for each receptor in the pair, as the fraction of permutations in the null distribution that have a correlation greater in magnitude than the correlation between the DN model constant and the two-dimensional GLM with both true receptor densities in the pair. This procedures provides the probability that the correlation coefficient brought about by the two-receptor models could also have been obtained by combining one of the two receptors with a surrogate receptor, with the same distribution of values as the other receptor in the pair but randomized spatial structure.

### ROI definition

We use ROIs defined by the HCP-MMP atlas (*42*).

## Supplementary Materials

### This PDF file includes:

Figs. S1 to S8 Note S1

## References

[1] D. J. Heeger, M. Behrmann, I. Dinstein, Vision as a beachhead. Biol. Psychiatry 81, 832–837 (2017). 27884424 10.1016/j.biopsych.2016.09.019

[2] D. J. Heeger, Normalization of cell responses in cat striate cortex. Vis. Neurosci. 9, 181–197 (1992). 1504027 10.1017/s0952523800009640

[3] N. C. Rabinowitz, B. D. Willmore, J. W. Schnupp, A. J. King, Contrast gain control in auditory cortex. Neuron 70, 1178–1191 (2011). 21689603 10.1016/j.neuron.2011.04.030 PMC3133688

[4] G. J. Brouwer, V. Arnedo, S. Offen, D. J. Heeger, A. C. Grant, Normalization in human somatosensory cortex. J. Neurophysiol. 114, 2588–2599 (2015). 26311189 10.1152/jn.00939.2014 PMC4637367

[5] B. M. Harvey, M. J. Vansteensel, C. H. Ferrier, N. Petridou, W. Zuiderbaan, E. J. Aarnoutse, M. G. Bleichner, H. C. Dijkerman, M. J. E. van Zandvoort, F. S. S. Leijten, N. F. Ramsey, S. O. Dumoulin, Frequency specific spatial interactions in human electrocorticography: V1 alpha oscillations reflect surround suppression. Neuroimage 65, 424–432 (2013). 23085107 10.1016/j.neuroimage.2012.10.020

[6] J. F. Linden, R. C. Liu, M. Sahani, C. E. Schreiner, M. M. Merzenich, Spectrotemporal structure of receptive fields in areas AI and AAF of mouse auditory cortex. J. Neurophysiol. 90, 2660–2675 (2003). 12815016 10.1152/jn.00751.2002

[7] J. H. Reynolds, D. J. Heeger, The normalization model of attention. Neuron 61, 168–185 (2009). 19186161 10.1016/j.neuron.2009.01.002 PMC2752446

[8] K. Louie, T. LoFaro, R. Webb, P. W. Glimcher, Dynamic divisive normalization predicts time-varying value coding in decision-related circuits. J. Neurosci. 34, 16046–16057 (2014). 25429145 10.1523/JNEUROSCI.2851-14.2014 PMC4244470

[9] W. Schellekens, N. Petridou, N. F. Ramsey, Detailed somatotopy in primary motor and somatosensory cortex revealed by Gaussian population receptive fields. Neuroimage 179, 337–347 (2018). 29940282 10.1016/j.neuroimage.2018.06.062 PMC6413921

[10] M. Chirimuuta, Minimal models and canonical neural computations: The distinctness of computational explanation in neuroscience. Synthese 191, 127–153 (2014).

[11] M. Carandini, D. J. Heeger, Normalization as a canonical neural computation. Nat. Rev. Neurosci. 13, 51–62 (2012). 10.1038/nrn3136 PMC3273486 22108672

[12] M. Aqil, T. Knapen, S. O. Dumoulin, Divisive normalization unifies disparate response signatures throughout the human visual hierarchy. Proc. Natl. Acad. Sci. U.S.A. 118, (2021). 10.1073/pnas.2108713118 PMC8609633 34772812

[13] A. Plebe, The search of canonical explanations for the cerebral cortex. Hist. Philos. Life Sci. 40, 1–36 (2018). 10.1007/s40656-018-0205-2 29905901

[14] S. O. Dumoulin, B. A. Wandell, Population receptive field estimates in human visual cortex. Neuroimage 39, 647–660 (2008). 17977024 10.1016/j.neuroimage.2007.09.034 PMC3073038

[15] S. O. Dumoulin, T. Knapen, How visual cortical organization is altered by ophthalmologic and neurologic disorders. Annu. Rev. Vis. Sci. 4, 357–379 (2018). 29889657 10.1146/annurev-vision-091517-033948

[16] K. Zilles, N. Palomero-Gallagher, A. Schleicher, Transmitter receptors and functional anatomy of the cerebral cortex. J. Anat. 205, 417–432 (2004). 15610391 10.1111/j.0021-8782.2004.00357.x PMC1571403

[17] S. B. Eickhoff, C. Rottschy, M. Kujovic, N. Palomero-Gallagher, K. Zilles, Organizational principles of human visual cortex revealed by receptor mapping. Cereb. Cortex 18, 2637–2645 (2008). 18321873 10.1093/cercor/bhn024 PMC2733321

[18] A. Goulas, J. Changeux, K. Wagstyl, K. Amunts, N. Palomero-Gallagher, C. Hilgetag, The natural axis of transmitter receptor distribution in the human cerebral cortex. Proc. Natl. Acad. Sci. U.S.A. 118, (2021). 10.1073/pnas.2020574118 PMC7826352 33452137

[19] J. Y. Hansen, G. Shafiei, R. D. Markello, K. Smart, S. M. L. Cox, M. Nørgaard, V. Beliveau, Y. Wu, J. D. Gallezot, E. Aumont, S. Servaes, S. G. Scala, J. M. DuBois, G. Wainstein, G. Bezgin, T. Funck, T. W. Schmitz, R. N. Spreng, M. Galovic, M. J. Koepp, J. S. Duncan, J. P. Coles, T. D. Fryer, F. I. Aigbirhio, C. J. McGinnity, A. Hammers, J. P. Soucy, S. Baillet, S. Guimond, J. Hietala, M. A. Bedard, M. Leyton, E. Kobayashi, P. Rosa-Neto, M. Ganz, G. M. Knudsen, N. Palomero-Gallagher, J. M. Shine, R. E. Carson, L. Tuominen, A. Dagher, B. Misic, Mapping neurotransmitter systems to the structural and functional organization of the human neocortex. Nat. Neurosci. 14, 775–782 (2022). 10.1038/s41593-022-01186-3 PMC9630096 36303070

[20] A. Watakabe, Y. Komatsu, O. Sadakane, S. Shimegi, T. Takahata, N. Higo, S. Tochitani, T. Hashikawa, T. Naito, H. Osaki, H. Sakamoto, M. Okamoto, A. Ishikawa, Enriched expression of serotonin 1b and 2a receptor genes in macaque visual cortex and their bidirectional modulatory effects on neuronal responses. Cereb. Cortex 19, 1915–1928 (2009). 19056862 10.1093/cercor/bhn219 PMC2705701

[21] S. Shimegi, A. Kimura, A. Sato, C. Aoyama, R. Mizuyama, K. Tsunoda, F. Ueda, S. Araki, R. Goya, H. Sato, Cholinergic and serotonergic modulation of visual information processing in monkey v1. J. Physiol. 110, 44–51 (2016). 10.1016/j.jphysparis.2016.09.001 27619519

[22] L. Seillier, C. Lorenz, K. Kawaguchi, T. Ott, A. Nieder, P. Pourriahi, H. Nienborg, Serotonin decreases the gain of visual responses in awake macaque v1. J. Neurosci. 37, 11390–11405 (2017). 29042433 10.1523/JNEUROSCI.1339-17.2017 PMC5700422

[23] Z. Azimi, R. Barzan, K. Spoida, T. Surdin, P. Wollenweber, M. D. Mark, S. Herlitze, D. Jancke, Separable gain control of ongoing and evoked activity in the visual cortex by serotonergic input. eLife 9, e53552 (2020). 32252889 10.7554/eLife.53552 PMC7138610

[24] S. Katzner, L. Busse, M. Carandini, GABAA inhibition controls response gain in visual cortex. J. Neurosci. 31, 5931–5941 (2011). 21508218 10.1523/JNEUROSCI.5753-10.2011 PMC3083851

[25] A. A. Disney, Neuromodulatory control of early visual processing in macaque. Annu. Rev. Vis. Sci. 7, 181–199 (2021). 34524875 10.1146/annurev-vision-100119-125739

[26] D. A. Lewis, J. N. Pierri, D. W. Volk, D. S. Melchitzky, T. U. W. Woo, Altered gaba neurotransmission and prefrontal cortical dysfunction in schizophrenia. Biol. Psychiatry 46, 616–626 (1999). 10472415 10.1016/s0006-3223(99)00061-x

[27] D. S. Schwarzkopf, E. J. Anderson, B. de Haas, S. J. White, G. Rees, Larger extrastriate population receptive fields in autism spectrum disorders. J. Neurosci. 34, 2713–2724 (2014). 24523560 10.1523/JNEUROSCI.4416-13.2014 PMC3921434

[28] E. J. Anderson, M. S. Tibber, D. S. Schwarzkopf, S. S. Shergill, E. Fernandez-Egea, G. Rees, S. C. Dakin, Visual population receptive fields in people with schizophrenia have reduced inhibitory surrounds. J. Neurosci. 37, 1546–1556 (2017). 28025253 10.1523/JNEUROSCI.3620-15.2016 PMC5299570

[29] G. Northoff, H. Mushiake, Why context matters? Divisive normalization and canonical microcircuits in psychiatric disorders. Neurosci. Res. 156, (2020). 10.1016/j.neures.2019.10.002 31628970

[30] D. Marr, *Vision: A Computational Investigation into the Human Representation and Processing of Visual Information* (Henry Holt and Co. Inc., USA), (1982).

[31] V. Beliveau, M. Ganz, L. Feng, B. Ozenne, L. Højgaard, P. M. Fisher, C. Svarer, D. N. Greve, G. M. Knudsen, A high-resolution in vivo atlas of the human brain’s serotonin system. J. Neurosci. 37, 120–128 (2017). 28053035 10.1523/JNEUROSCI.2830-16.2016 PMC5214625

[32] V. Beliveau, B. Ozenne, S. Strother, D. N. Greve, C. Svarer, G. M. Knudsen, M. Ganz, The structure of the serotonin system: A pet imaging study. Neuroimage 205, 116240 (2020). 31600591 10.1016/j.neuroimage.2019.116240 PMC6951807

[33] M. Nørgaard, V. Beliveau, M. Ganz, C. Svarer, L. H. Pinborg, S. H. Keller, P. S. Jensen, D. N. Greve, G. M. Knudsen, A high-resolution in vivo atlas of the human brain’s benzodiazepine binding site of GABAA receptors. Neuroimage 232, 117878 (2021). 33610745 10.1016/j.neuroimage.2021.117878 PMC8256681

[34] N. C. Benson, K. W. Jamison, M. J. Arcaro, A. T. Vu, M. F. Glasser, T. S. Coalson, D. C. Van Essen, E. Yacoub, K. Ugurbil, J. Winawer, K. Kay, The Human Connectome Project 7 tesla retinotopy dataset: Description and population receptive field analysis. J. Vis. 18, 23–23 (2018). 10.1167/18.13.23 PMC6314247 30593068

[35] B. A. Wandell, J. Winawer, Computational neuroimaging and population receptive fields. Trends Cogn. Sci. 19, 349–357 (2015). 25850730 10.1016/j.tics.2015.03.009 PMC4484758

[36] P. C. Klink, X. Chen, W. Vanduffel, P. Roelfsema, Population receptive fields in nonhuman primates from whole-brain fMRI and large-scale neurophysiology in visual cortex. eLife 10, e67304 (2021). 34730515 10.7554/eLife.67304 PMC8641953

[37] W. Zuiderbaan, B. M. Harvey, S. O. Dumoulin, Modeling center–surround configurations in population receptive fields using fMRI. J. Vis. 12, 10–10 (2012). 10.1167/12.3.10 22408041

[38] K. N. Kay, J. Winawer, A. Mezer, B. A. Wandell, Compressive spatial summation in human visual cortex. J. Neurophysiol. 110, 481–494 (2013). 23615546 10.1152/jn.00105.2013 PMC3727075

[39] A. Angelucci, M. Bijanzadeh, L. Nurminen, F. Federer, S. Merlin, P. C. Bressloff, Circuits and mechanisms for surround modulation in visual cortex. Annu. Rev. Neurosci. 40, 425–451 (2017). 28471714 10.1146/annurev-neuro-072116-031418 PMC5697758

[40] M. F. Glasser, S. N. Sotiropoulos, J. A. Wilson, T. S. Coalson, B. Fischl, J. L. Andersson, J. Xu, S. Jbabdi, M. Webster, J. R. Polimeni, D. C. Van Essen, M. Jenkinson, The minimal preprocessing pipelines for the Human Connectome Project. Neuroimage 80, 105–124 (2013). 23668970 10.1016/j.neuroimage.2013.04.127 PMC3720813

[41] B. A. Wandell, S. O. Dumoulin, A. A. Brewer, Visual field maps in human cortex. Neuron 56, 366–383 (2007). 17964252 10.1016/j.neuron.2007.10.012

[42] M. F. Glasser, T. S. Coalson, E. C. Robinson, C. D. Hacker, J. Harwell, E. Yacoub, K. Ugurbil, J. Andersson, C. F. Beckmann, M. Jenkinson, S. M. Smith, D. C. Van Essen, A multi-modal parcellation of human cerebral cortex. Nature 536, 171–178 (2016). 27437579 10.1038/nature18933 PMC4990127

[43] E. Hendrikx, J. M. Paul, M. van Ackooij, N. van der Stoep, B. M. Harvey, Visual timing-tuned responses in human association cortices and response dynamics in early visual cortex. Nat. Commun. 13, 1–19 (2022). 35804026 10.1038/s41467-022-31675-9 PMC9270326

[44] J. Zhou, N. C. Benson, K. N. Kay, J. Winawer, Compressive temporal summation in human visual cortex. J. Neurosci. 38, 691–709 (2018). 29192127 10.1523/JNEUROSCI.1724-17.2017 PMC5777115

[45] T. Sawada, A. A. Petrov, The divisive normalization model of v1 neurons: A comprehensive comparison of physiological data and model predictions. J. Neurophysiol. 118, 3051–3091 (2017). 28835531 10.1152/jn.00821.2016 PMC5814712

[46] B. M. Harvey, S. O. Dumoulin, The relationship between cortical magnification factor and population receptive field size in human visual cortex: Constancies in cortical architecture. J. Neurosci. 31, 13604–13612 (2011). 21940451 10.1523/JNEUROSCI.2572-11.2011 PMC6623292

[47] P. Bednaˇrík, I. Tkác, F. Giove, M. DiNuzzo, D. K. Deelchand, U. E. Emir, L. E. Eberly, S. Mangia, Neurochemical and bold responses during neuronal activation measured in the human visual cortex at 7 tesla. J. Cereb. Blood Flow Metab. 35, 601–610 (2015). 25564236 10.1038/jcbfm.2014.233 PMC4420878

[48] D. B. Terhune, E. Murray, J. Near, C. J. Stagg, A. Cowey, Phosphene perception relates to visual cortex glutamate levels and covaries with atypical visuospatial awareness. Cereb. Cortex 25, 4341–4350 (2015). 25725043 10.1093/cercor/bhv015 PMC4816785

[49] F. C. Widmer, S. M. O’Toole, G. B. Keller, Nmda receptors in visual cortex are necessary for normal visuomotor integration and skill learning. eLife 11, e71476 (2022). 35170429 10.7554/eLife.71476 PMC8901170

[50] V. J. Li, A. Schohl, E. S. Ruthazer, Topographic map formation and the effects of nmda receptor blockade in the developing visual system. Proc. Natl. Acad. Sci. U.S.A. 119, e2107899119 (2022). 35193956 10.1073/pnas.2107899119 PMC8872792

[51] M. Aqil, T. Knapen, Prfpy: A Python package to simulate and fit population receptive field models to time series data. (v0.1.0-alpha). Zenodo. (2023); 10.5281/zenodo.10201022.

[52] M. Aqil, Prfpytools: A Python package for analysis and visualization of population receptive field models. (v0.1.0-alpha). Zenodo. (2023); 10.5281/zenodo.10209657.

