# Supplementary material for: Computational model links normalization to chemoarchitecture in the human visual system
Source: Sci Adv. 2024 Jan 3;10(1):eadj6102. doi: 10.1126/sciadv.adj6102 (PMC10776006; doi:10.1126/sciadv.adj6102)
Supplement: Supplementary file 1 — Figs. S1 to S8 Note S1 [file sciadv.adj6102_sm.pdf]

Science Advances
AAAS

Supplementary Materials for

Computational model links normalization to chemoarchitecture in the human visual system

Marco Aqil *et al.*

Corresponding author: Marco Aqil, m.aqil@spinozacentre.nl

Sci. Adv. **10**, eadj6102 (2024)
DOI: 10.1126/sciadv.adj6102

This PDF file includes:

Figs. S1 to S8
Note S1

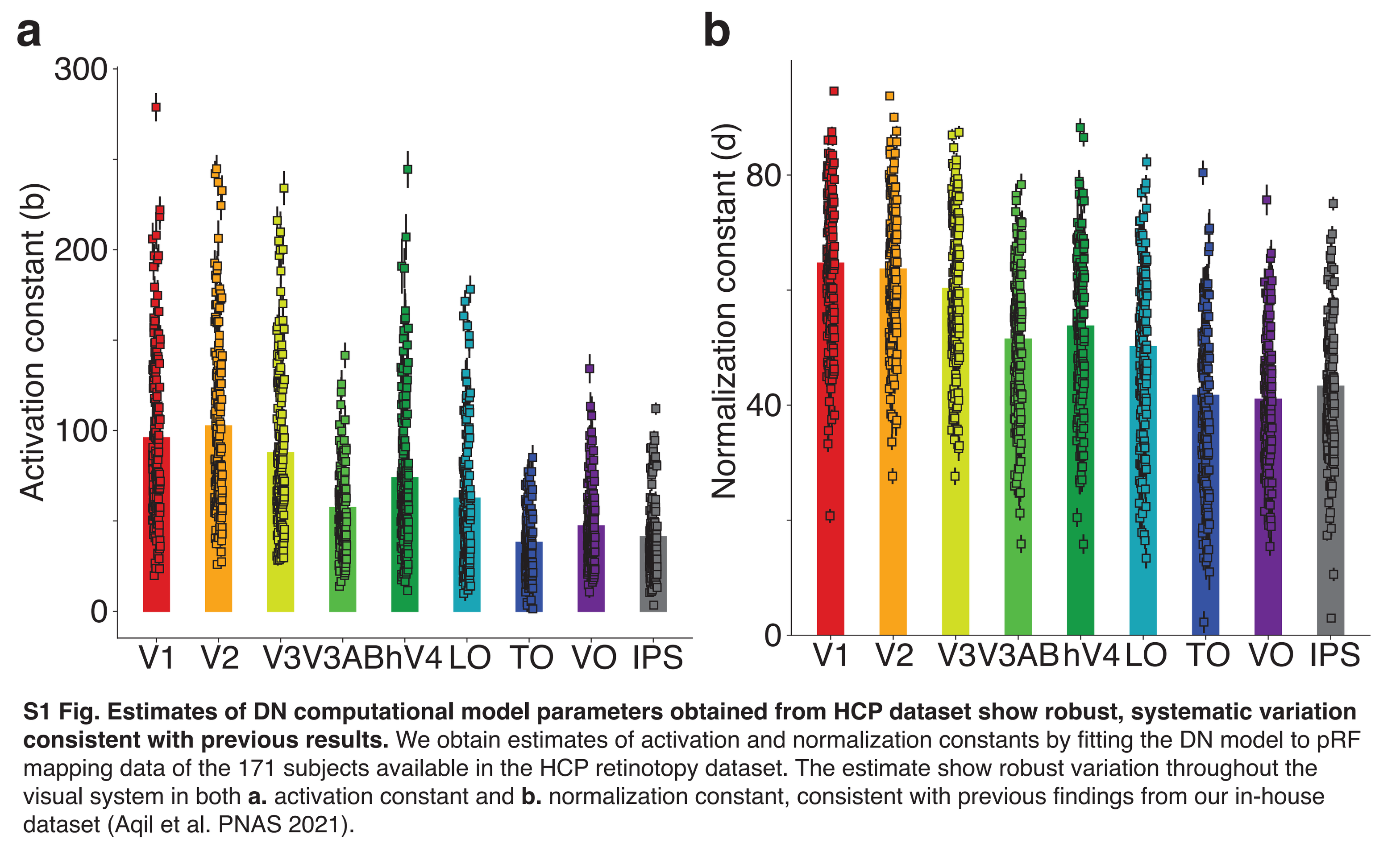

S1 Fig. Estimates of DN computational model parameters obtained from HCP dataset show robust, systematic variation consistent with previous results. We obtain estimates of activation and normalization constants by fitting the DN model to pRF mapping data of the 171 subjects available in the HCP retinotopy dataset. The estimate show robust variation throughout the visual system in both **a.** activation constant and **b.** normalization constant, consistent with previous findings from our in-house dataset (Aqil et al. PNAS 2021).

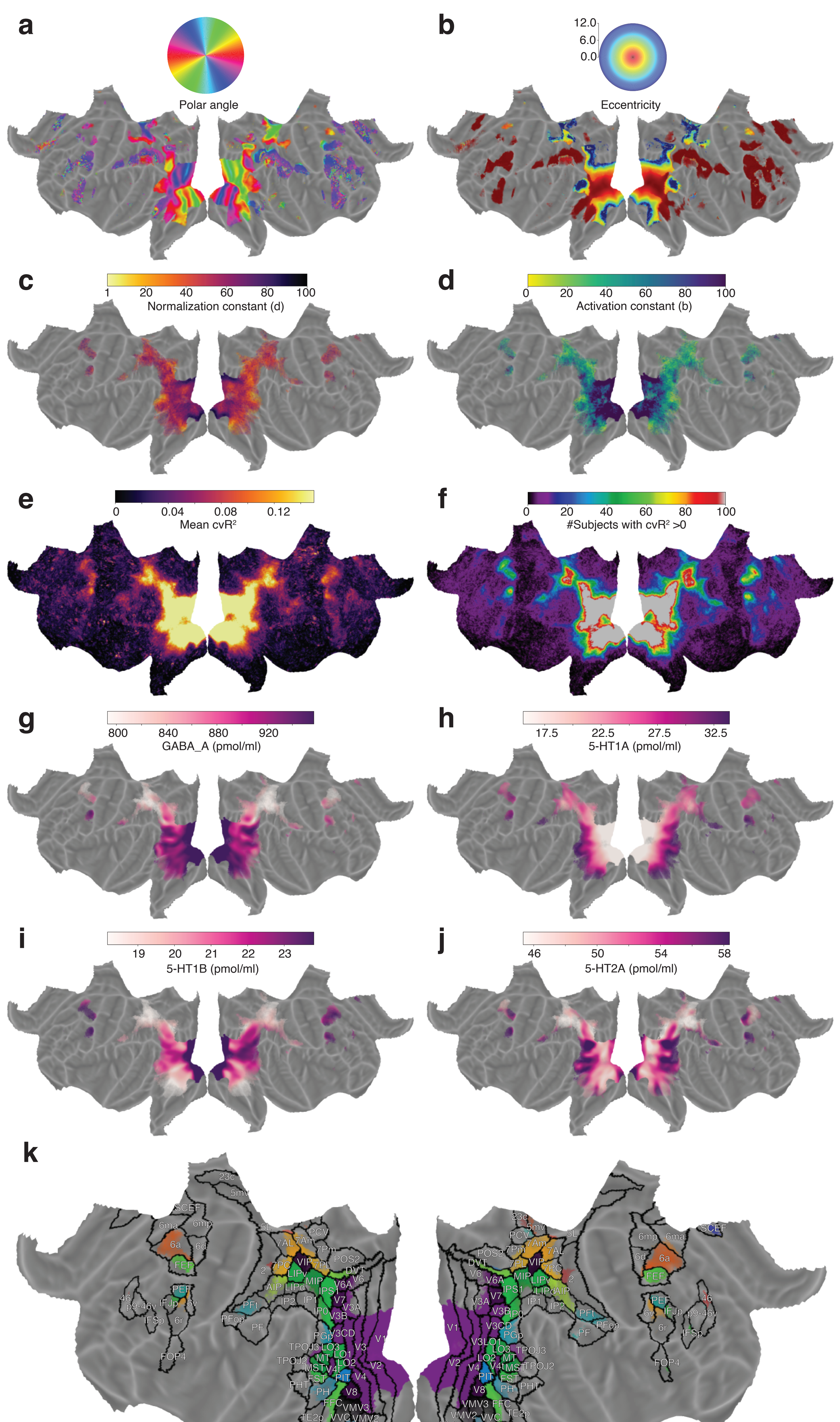

S2 Fig. Flatmaps of DN computational model parameters, receptor density maps, and Glasser-HCP Atlas ROIs. a,b. Polar angle and eccentricity maps obtained by replicating the HCP retinotopy pRF analysis pipeline, with the only difference being that we use the DN model instead of CSS. The resulting maps show that the DN model recovers well-known visual field maps throughout the visual system. **c,d.** Population-average cortical maps of DN activation and normalization constants. **e.** Population-average crossvalidated variance explained (cvR²) of the DN model. **f.** Number of HCP subjects with cvR² >0 at each cortical location. **g,h,i,j.** Relevant rceptor density maps obtained by PET imaging datasets. **k.** ROIs included in analysis. Defined as all Glasser-HCP ROIs with at least 10 vertices where the number of participants with cvR² >0 is above the Li cross-entropy threshold (27 participants).

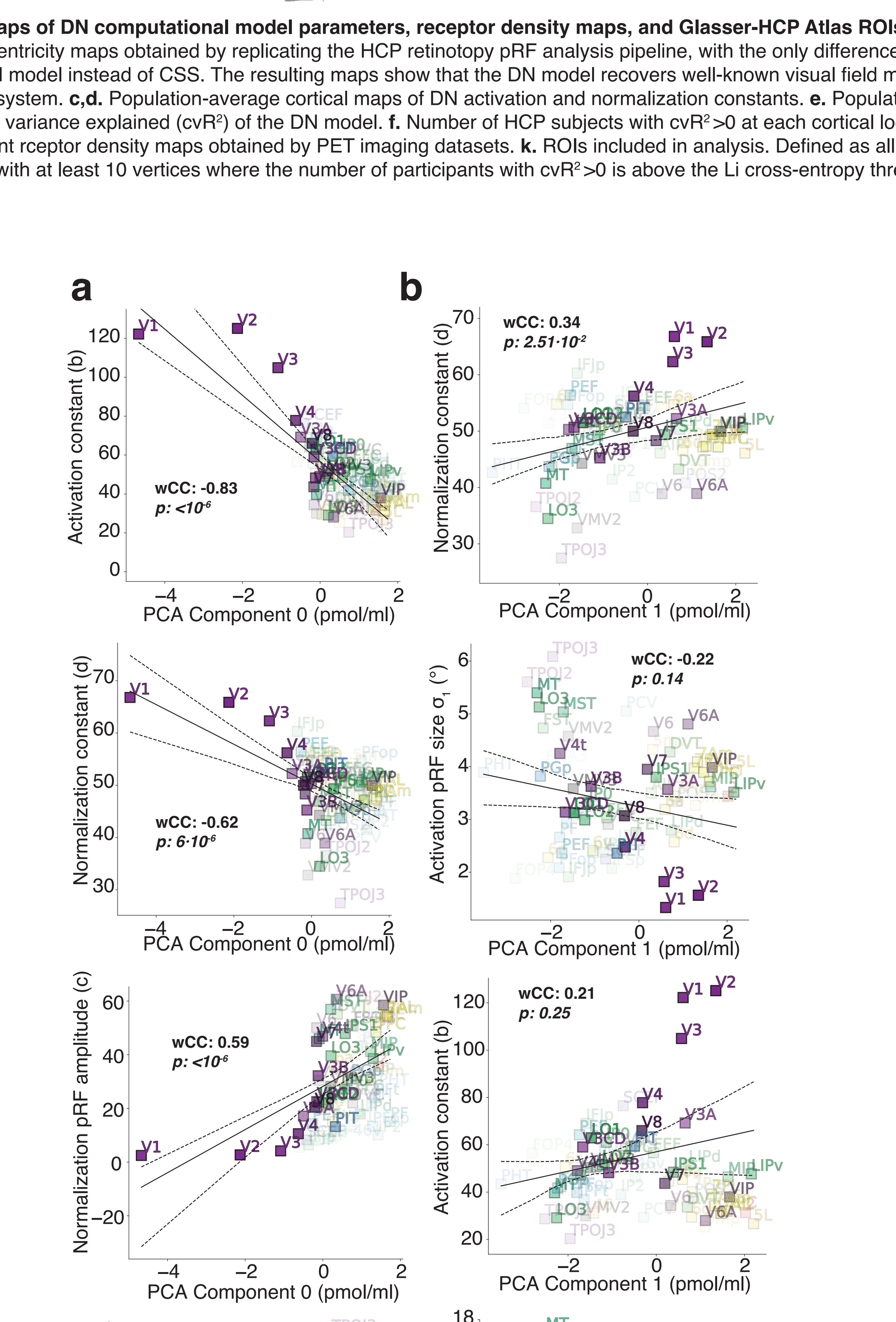

S3 Fig. Correlations between DN model parameters and receptor dataset PCA components. We show correlations and p-values between DN model parameters and the first two PCA components of the receptor dataset, sorted top to bottom by strenght of the correlation. **a.** The first PCA component of the receptor dataset correlates most strongly with the DN model activation constant. **b.** The second component correlates most strongly with the DN model normalization constant.

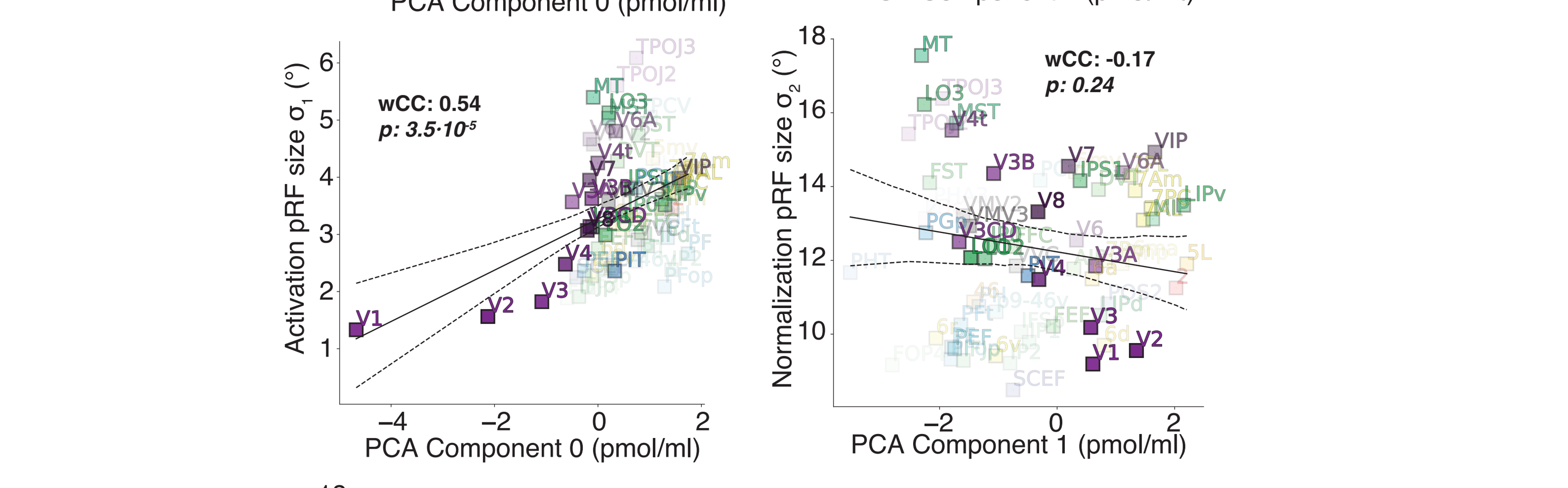

S4 Fig. Correlations between DN model parameters and receptor densities in the absence of V1. V1 is an ROI with special biological and computational signficance, and indeed presents some of the highest estimates both for receptor densities and DN computational model parameters. To investigate the influence of V1 on the hypothesised correlations, we recompute them without this ROI. We find that, in the absence of V1 **a.** the correlations of GABA_A and 5-HT1A densities with the activation constant remain highly significant. **b.** The hypothesised correlation of 5-HT1B and 5-HT2A density with the normalization constant are n.s.. See S5 Fig for the 2D-GLM analysis recomputed in the absence of V1.

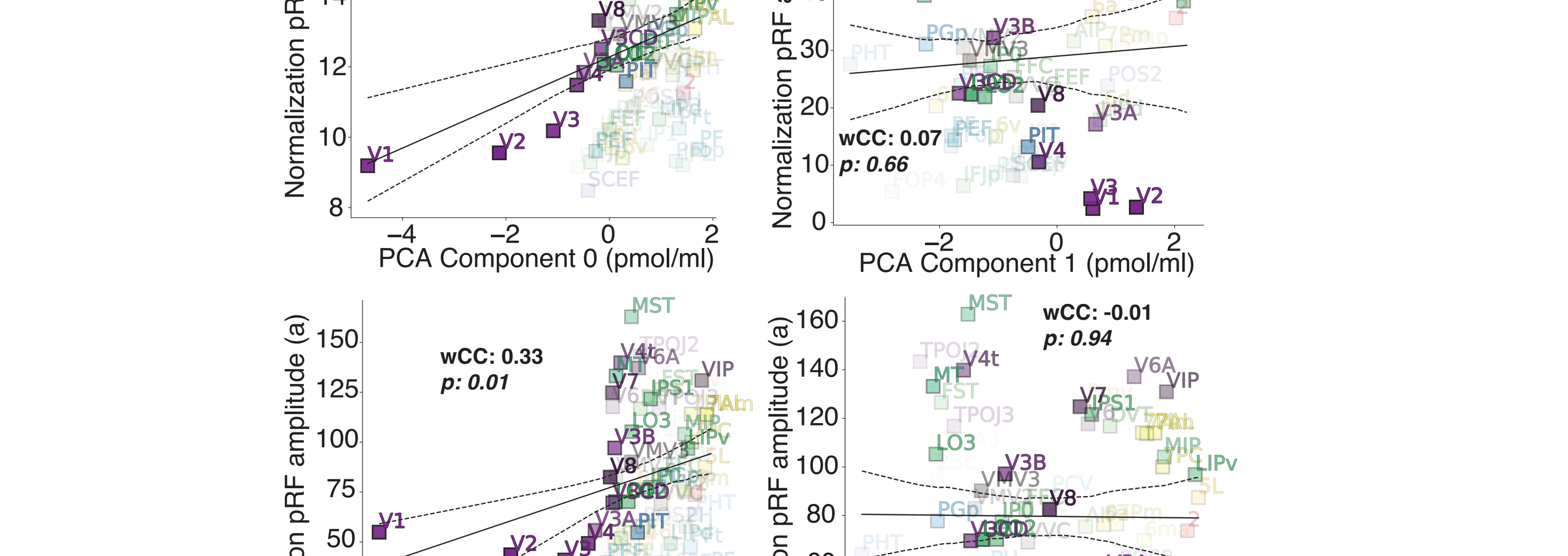

S5 Fig. 2D-GLM correlations between DN model parameters and receptor densities in the absence of V1. To investigate the extent to which the identified correlations are influenced by V1, we recompute the 2D-GLM analysis in the absence of V1. The increases in crossvalidated correlation coefficient wCC(cv) brought about by 2-receptor models, and partial correlations of each receptor, persist in the absence of V1, showing that the pairs of receptors maintain notable relationships with DN model computational parameters throughout the human visual system, even in the absence of V1. **a.** 2D-GLM of GABA_A and 5-HT1A on DN model activation constant, and crossvalidated correlation coefficient for individual and combined receptors. **b.** 2D-GLM of 5-HT1B and 5-HT2A on DN model normalization constant, and crossvalidated correlation coefficient for individual and combined receptors. **c.** Partial correlations of GABA_A and 5-HT1A on DN model activation constant and respective p-values. **d.** Partial correlations of 5-HT1B and 5-HT2A on DN model normalization constant and respective p-values (see Methods for the p-values computation). In brief, the p-values reported for each receptor give the probability that a correlation of equivalent or greater magnitude as the true correlation could also have been obtained by a surrogate 2D-GLM containing the opposite receptor in the pair, and a randomized permutation of the receptor under investigation.

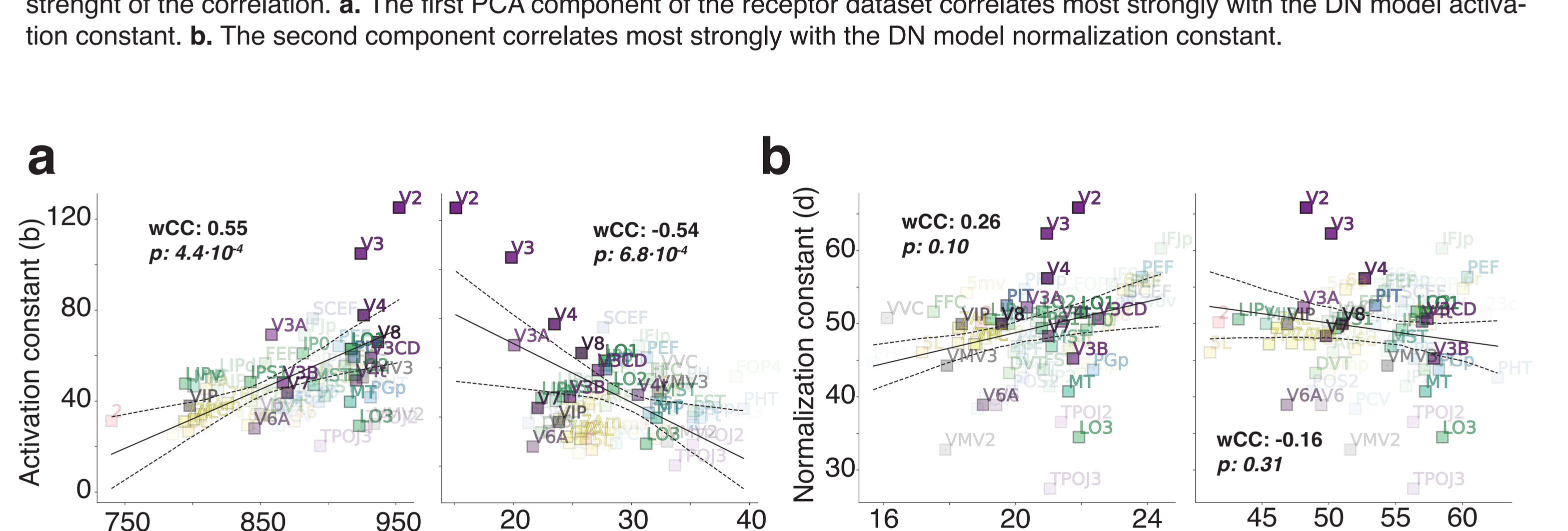

S6 Fig. Correlations between DN model parameters and receptor densities on unparcellated data. To investigate whether ROI parcellation has an influence on our results, we also compute the correlations on the full vertex-wise unparcellated data. In each plot, the red hexbins show the density of datapoints at each location; the black squares the ROI-means; the black line represents the correlation fit on the ROI-means; the white line the correlation fit on the unparcellated data. The inset reports the correlation coefficient and the p-value on the unparcellated data. To compute each p-value while taking into account the specific spatial autocorrelations present in the datasets, we use a graph-eigenmode approach (see S1 Supplementary Note). **a.** The correlations of GABA_A and 5-HT1A densities, positive and negative respectively, with the activation constant remain strongly significant. **b.** The positive correlation of 5-HT1B density with the normalization constant remains strongly significant, while the correlation of 5-HT2A density is n.s. (as in the ROI-means case, where this correlation was only significant in the 2D-GLM approach).

S1 Supplementary Note. Computing null-distributions for unparcellated data. To test the statistical significance of correlations in the unparcellated (vertex-wise) data, we need to compute null-distributions for each receptor. A naive way to obtain such null-distributions is computing a large number of permutations, each obtained by randomizing the vertex labels in the dataset. However, this approach does not preserve the spatial fingerprint (i.e. autocorrelation) of the data and hence, because of the high number of datapoints, results in 'statistically significant' results even for very weak correlations. To address this concern and estimate statistical significance on unparcellated note in a more conservative way, we compute null-distributions that preserve the specific spatial fingerprint of each receptor, while randomizing the location of its patterns. To do this, we employ methods from graph theory. In essence, the procedure is the same as methods commonly employed in the temporal analysis of time series data, which randomize the Fourier phase of a signal, while preserving its power spectrum. The only difference is that we do this in space (or more specifically, on the graph defined by a cortical surface mesh) rather than in time. We first construct a graph adjacency matrix including all the vertices on the fsaverage cortical mesh where we have datapoints (i.e. the colored vertices visible on Fig. 3 and S2 Fig., see also Methods). Next, we compute the graph Laplacian of this graph, and taking advantage of the fact that the graph Laplacian eigenvectors implement the graph Fourier transform, we compute the graph-Fourier transform of each receptor density pattern. Next, we compute 10^6 permutations to obtain each receptor's specific null-distribution by randomizing the sign of each eigenmode coefficient. This procedure ensures that each permutation has (approximately) the same spatial fingerprint as the original receptor, but randomized locations of where patterns occur. Finally, we recompute the correlation for each permutation in the null-distribution, and obtain the two-sided p-value as the proportion of times that a randomized permutation had a correlation higher in magnitude than the true dataset. This procedure allows us to test the hypothesis that a receptor with similar spatial structure to the 'true' one could also have produced a similar correlation.

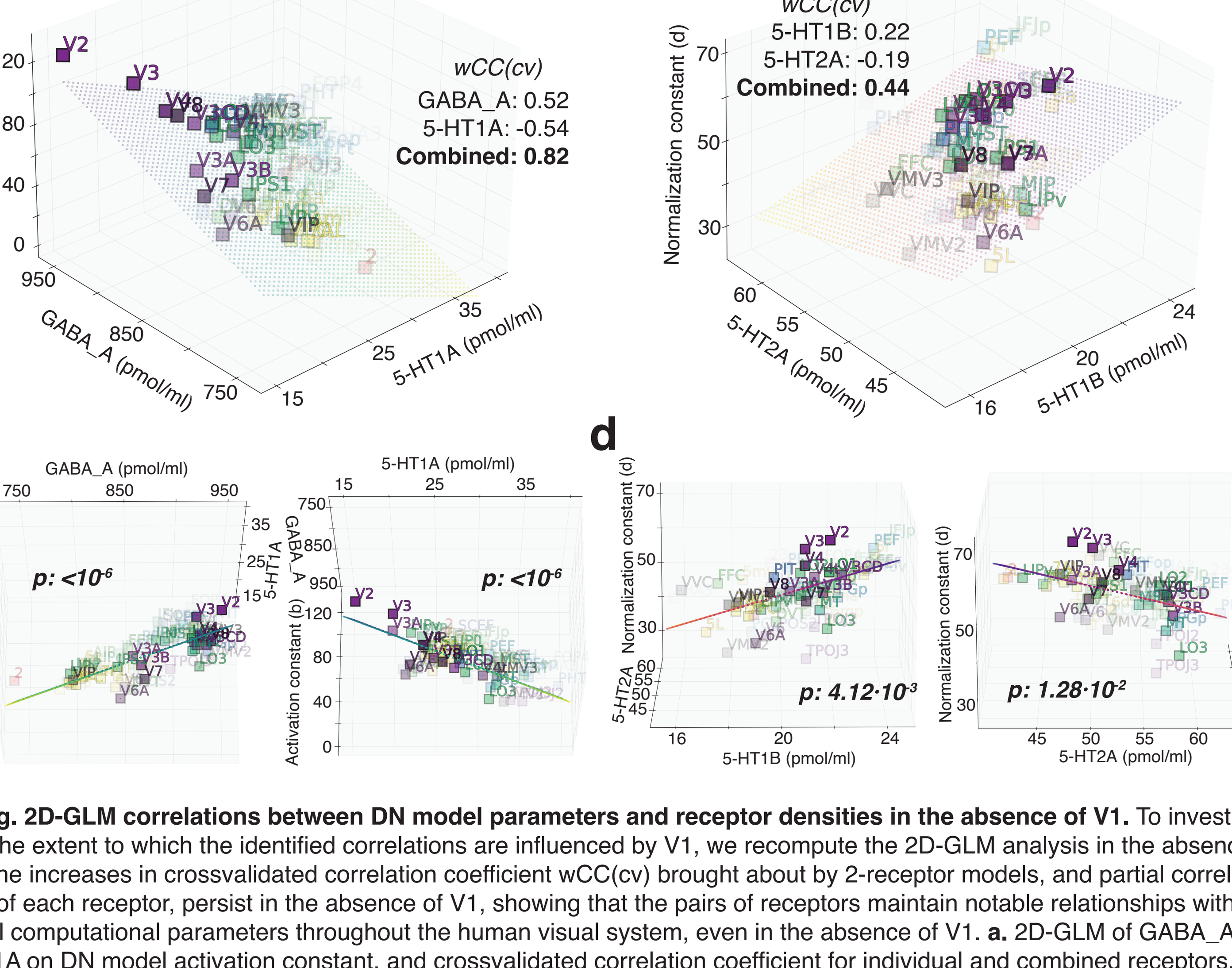

S7 Fig. Correlations between activation constant in alternative model formulation (d=1) and relevant receptor densities. The estimates of the activation constant in a reduced model with d=1 can be mathematically obtained by dividing the estimate of b in the original formulation by the corresponding value of d. We find that the estimates of activation constant in the d=1 model (i.e. b/d in the original formulation) still correlate with GABA and 5-HT1A receptor densities, consistent with our hypothesis. Correlations of the normalization constant with 5-HT1B and 5-HT2A cannot be obtained, since the parameter is kept fixed to d=1 in this formulation.

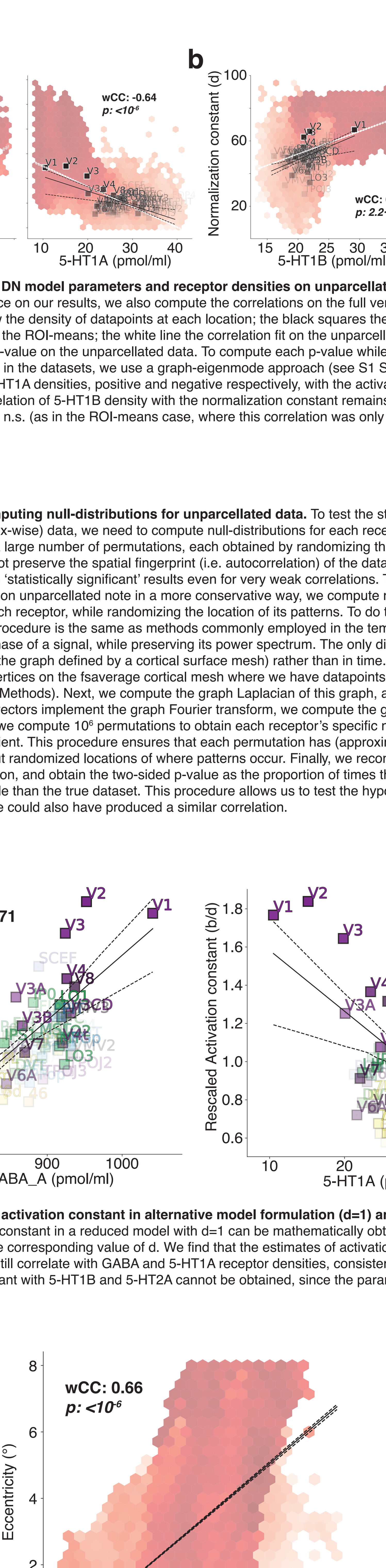

S8 Fig. Serotonin transporter (5-HTT) receptor density significantly correlates with visual eccentricity. We find a statistically significant and robust correlation between the serotonin transporter (5-HTT) receptor density and visual eccentricity throughout the visual system. The correlation and p-value (computed following S1 Supplementary Note) are shown for the unparcellated data, since eccentricity primarily varies within-ROIs, and the "mean eccentricity" of an ROI is not well-defined. This finding was not part of our hypotheses, and hence we do not include it in our main results. We report it here since, to the best of our knowledge, this is a novel finding in humans, and might be of potential functional relevance and worth of future investigation.
